# A novel *MBTPS2* variant associated with BRESHECK syndrome impairs sterol‐regulated transcription and the endoplasmic reticulum stress response

**DOI:** 10.1002/ajmg.a.62537

**Published:** 2021-10-15

**Authors:** Alanna Strong, Michael E. March, Christopher J. Cardinale, Sophia E. Kim, Jamie Merves, Hilary Whitworth, Leslie Raffini, Christopher Larosa, Lawrence Copelovitch, Cuiping Hou, Diana Slater, Courtney Vaccaro, Deborah Watson, Elaine H. Zackai, Jeffrey Billheimer, Hakon Hakonarson

**Affiliations:** ^1^ Division of Human Genetics Children's Hospital of Philadelphia Philadelphia Pennsylvania USA; ^2^ The Center for Applied Genomics Children's Hospital of Philadelphia Philadelphia Pennsylvania USA; ^3^ Division of Gastroenterology, Hepatology and Nutrition Children's Hospital of Philadelphia Philadelphia Pennsylvania USA; ^4^ Department of Pediatrics, Perelman School of Medicine University of Pennsylvania Philadelphia Pennsylvania USA; ^5^ Division of Hematology Children's Hospital of Philadelphia Philadelphia Pennsylvania USA; ^6^ Division of Nephrology Children's Hospital of Philadelphia Philadelphia Pennsylvania USA; ^7^ Division of Translational Medicine and Human Genetics, Department of Medicine University of Pennsylvania Philadelphia Pennsylvania USA; ^8^ Division of Pulmonary Medicine Children's Hospital of Philadelphia Philadelphia Pennsylvania USA

**Keywords:** BRESHECK syndrome, cholesterol, ER stress, ichthyosis follicularis with atrichia photophobia syndrome, *MBTPS2*

## Abstract

Ichthyosis follicularis, atrichia, and photophobia syndrome (IFAP syndrome) is a rare, X‐linked disorder caused by pathogenic variants in membrane‐bound transcription factor protease, site 2 (*MBTPS2*). Pathogenic *MBTPS2* variants also cause BRESHECK syndrome, characterized by the IFAP triad plus intellectual disability and multiple congenital anomalies. Here we present a patient with ichthyosis, sparse hair, pulmonic stenosis, kidney dysplasia, hypospadias, growth failure, thrombocytopenia, anemia, bone marrow fibrosis, and chronic diarrhea found by research‐based exome sequencing to harbor a novel, maternally inherited *MBTPS2* missense variant (c.766 G>A; (p.Val256Leu)). In vitro modeling supports variant pathogenicity, with impaired cell growth in cholesterol‐depleted media, attenuated activation of the sterol regulatory element‐binding protein pathway, and failure to activate the endoplasmic reticulum stress response pathway. Our case expands both the genetic and phenotypic spectrum of BRESHECK syndrome to include a novel *MBTPS2* variant and cytopenias, bone marrow fibrosis, and chronic diarrhea.

## 1 INTRODUCTION

Ichthyosis follicularis, atrichia, and photophobia (IFAP) Syndrome 1 with or without brain anomalies, retardation, ectodermal dysplasia, skeletal malformations, Hirschsprung disease, ear/eye anomalies, cleft palate/cryptorchidism, and kidney dysplasia/hypoplasia (BRESHECK) syndrome (OMIM #308205) is an X‐linked multiple malformation syndrome characterized by ichthyosis, photophobia, atrichia, intellectual disability, seizures, sensorineural hearing loss, cleft palate, congenital heart disease, renal dysplasia, cryptorchidism, skeletal abnormalities, failure to thrive, and Hirschsprung disease caused by hemizygous pathogenic variants in *MBTPS2* (Naiki et al., 2012; Reish et al., 1997). *MBTPS2* (membrane‐bound transcription factor protease, site 2), encodes site 2 protease (S2P), a zinc protease critical for sterol‐regulated gene transcription and activation of the endoplasmic reticulum (ER) stress response (Brown & Goldstein, 1997; Horton et al., 2002; Ye et al., 2000). Pathogenic *MBTPS2* variants also cause (1) IFAP syndrome, characterized by the clinical triad of ichthyosis, photophobia, and atrichia (Oeffner et al., 2009); (2) keratosis follicularis spinulosa decalvans (KFSD), characterized by hyperkeratosis predominantly of the palms and soles, photophobia, and sparse hair (Aten et al., 2010); (3) Olmsted syndrome, characterized by mutilating palmoplantar keratoderma and alopecia (Haghighi et al., 2013); and (4) osteogenesis imperfecta (OI), characterized by fragile bones and recurrent fractures (Lindert et al., 2016).

Multiple *MBTPS2* variants are causal for each of the MBTPS2‐associated syndromes (Table 1); however, to date, only the Arg429His variant has been associated with BRESHECK syndrome (Naiki et al., 2012). Many identified causal variants have been studied in vitro and demonstrate impaired growth in cholesterol‐deficient media and impaired activation of the ER stress response (Aten et al., 2010; Bornholdt et al., 2013; Lindert et al., 2016; Oeffner et al., 2009). Here, we describe a patient with ichthyosis, pulmonic stenosis, renal dysplasia, hypospadias, growth failure, thrombocytopenia, anemia, bone marrow fibrosis, and chronic diarrhea found by research‐based exome sequencing to harbor a novel, maternally inherited Val256Leu *MBTPS2* variant. The Val256Leu variant was unable to activate SREBP signaling, allow cell viability in cholesterol‐depleted media, nor activate the ER stress response. We propose that the Val256Leu *MBTPS2* variant represents a second causal allele for BRESHECK syndrome with bone marrow fibrosis, cytopenias, and chronic diarrhea as new manifestations of the phenotype.

**TABLE 1:** Previously reported *MBTPS2* variants and associated phenotypes

Case number	cDNA position	Protein change	Phenotype	Domain
1	c.71 T > C	p.Leu24Pro	IFAP	TM1
2	c.225–6 T > A	Extends Exon Three	IFAP	
3	c.261 G > A	p.Met87Iso	IFAP	TM2
4	c.599 C > T	p.Ala200Val	KFSD	TM4
5	c.638 C > T	p.Ser213Leu	KFSD	TM4
6	c.671–9 T > G	p.Iso225Leufs*25	IFAP	TM4
7	c.671–9 T > G	p.Iso225Leufs*25	Olmsted syndrome	TM4
8	c.667 G > T	p.Trp226Leu	IFAP	TM4
9	c.680 A > T	p.His227Leu	IFAP	TM4
10	c.686 T > C	p.Phe229Ser	IFAP	TM4
11	c.758 G > C	p.Gly253Ala	IFAP	TM5
**12**	**c.766 G > T**	**p.Val256Leu**	**Bresheck syndrome**	**TM5**
13	c.774 C > G	p.Iso258Met	IFAP	TM5
14	c.1001 G > A	p.Cys334Tyr	IFAP	Luminal
15	c.1286 G > A	p.Arg429His	IFAP	TM6
16	c.1286 G > A	p.Arg429His	Bresheck syndrome	TM6
17	c.1298 T > C	p.Leu433Pro	IFAP	TM6
18	c.1360 G > C	p.Ala454Pro	IFAP	TM7
19	c.1376 A > G	p.Asn459Ser	Osteogenesis imperfecta	TM7
20	c.1391 T > C	p.Phe464Ser	Olmsted syndrome	TM7
21	c.1424 T > C	p.Phe475Ser	IFAP	TM7
22	c.1427 T > C	p.Leu476Ser	IFAP	TM7
23	c.1430 A > T	p.Asp477Val	IFAP	TM7
24	c.1433 C > A	p.Ala478Asp	IFAP	Luminal
25	c.1494 G > T	p.Leu498Phe	IFAP	TM8
26	c.1499 G > A	p.Gly500Asp	KFSD	TM8
27	c.1499 G > A	p.Gly500Asp	IFAP	TM8
28	c.1515 G > C	p.Leu505Phe	Osteogenesis imperfecta	TM8
29	c.1523 A > G	p.Asn508Ser	KFSD	TM8
30	c.1523 A > G	p.Asn508Ser	IFAP	TM8
31	c.1523 A > C	p.Asn508Thr	IFAP	TM8
32	c.1538 T > C	p.Leu513Pro	IFAP	TM8

## 2 METHODS

### 2.1 Genetic testing methodology

Genotyping microarray and exome sequencing were performed at Columbia University. Clinical exome reanalysis was also performed at Columbia University. Mitochondrial DNA sequencing and deletion/duplication analysis was done at The Children's Hospital of Philadelphia. This testing was nondiagnostic. Clinical confirmation of the identified *MBTPS2* variant identified on research‐based exome sequencing was performed by PreventionGenetics.

### 2.2 Research‐based exome sequencing

Variant annotation, filtration, and prioritization were performed with GDCross, a variant annotation and prioritization platform developed within the Center for Applied Genomics (CAG). Variants with ≥5× coverage were initially filtered at 0.5% gnomAD mean allele frequency and annotated with a combination of multiple tools and databases, including Variant Effect Predictor, HGMD, ClinVar, dbSNP, OMIM, HPO, PolyPhen‐2, and SIFT, and a custom‐built splice‐site annotator. The list of patient variants was filtered against the pedigree and HPO terms describing the patient's phenotype, and GDCross assigned each variant a priority score of likelihood as the causal variant for the patient's disease. Variants were ranked using a weighted combination of multiple factors, including (a) overlap with HPO terms; (b) patient and family genotypes; (c) predicted functional impact; (d) inheritance modeling; and (e) presence in mutation databases such as HGMD and ClinVar. Research‐based exome sequencing was notable for a maternally inherited variant of uncertain significance in *MBTPS2* (c.766 G>A; p.Val256Leu). Clinical validation was performed at PreventionGenetics.

### 2.3 Cell culture

M19‐CHO cells (Hasan et al., 1994; Rawson et al., 1997), a mutant cell line of CHO‐K1 that are auxotrophic for cholesterol and fatty acids due to *MBTPS2* deficiency, were a generous gift from T.Y. Chang (Dartmouth Medical School, Hanover, NH, USA). Cells were propagated in DMEM:F12 media (Gibco) supplemented with 10% fetal bovine serum (Avantor) under standard conditions of 37°C and 5% CO_2_. Antibiotics were not added to the culture media.

### 2.4 Recombinant plasmids

Human *MBTPS2* cDNA in the pCMV backbone was purchased from Origene (RC218791). Mutations were introduced using the New England Biolabs Q5 polymerase system. The following primers were used at an annealing temperature of 60 degrees: F— TGGAGTTGGGTTGCTCATCAC, R—GTGTAGTAAAATGGCAAGAG (Val256Leu); F— TTTATCCCACATTTTAACTTTCTAAGC, R—ACTGGTGATGCTCACTGT (Arg429His); F— ATCTTGCTGGATGGCAGTGTA, R—GAAAAACCCTATTAGATCTTTGAC (Gly500Asp). To subclone into the pcDNA3 vector, the following primers were used to add a 5′‐Kozak sequence, 5′ BamHI site, and a 3′‐EcoRI site, respectively: F—GCATGGATCCGCCACCATGATTCCGGTGTCG, R—CGATGAATTCGCCGTTTAAACCTTATCGTC. The polymerase chain reaction (PCR) products and pcDNA3 vector were digested in parallel with BamHI and EcoRI and ligated using the New England Biolabs quick ligation kit. All mutations were confirmed by Sanger sequencing.

### 2.5 Creation of stable cell lines

*Mbtps2*‐deficient CHO cells (M19‐CHO) were plated in DMEM:F12 with 10% fetal bovine serum in 5.5‐cm dishes at a density of 1 × 10^5^ cells per dish. On Day 1, cells were transfected with 2 μg of pcDNA (control), wild‐type *MBTPS2* in pcDNA, or Val256Leu, Arg429His, or Gly500Asp variant‐*MBTPS2*. FuGENE HD (Promega) was used as the transfection reagent according to the manufacturer's protocol. On Day 3, media was changed to DMEM:F12 with 10% fetal bovine serum supplemented with 400 μg/ml of G418 (Corning). Media was changed every 3 days for 10 days. Cell lines were screened for *MBTPS2* expression via quantitative PCR and immunoblot.

### 2.6 Quantitative PCR

RNA was extracted from CHO‐M19 cells stably expressing pcDNA, WT‐*MBTPS2*, Val256Leu‐*MBTPS2*, Arg429His‐*MBTPS2*, or Gly500Asp‐*MBTPS2* using Trizol (QIAGEN). RNA was reverse transcribed into cDNA using the High Capacity cDNA Reverse Transcription Kit (Thermo Fisher Scientific). *MBTPS2* and GAPDH were amplified using TaqMan assay primers Hs00210639 and Hs02786624 and TaqMan Fast Advanced Master Mix according to the manufacturer's protocols (Thermo Fisher Scientific). BiP and actin were amplified using SYBR Green Master Mix (Thermo Fisher Scientific) according to the manufacturer's protocols. BiP was amplified using the primer pair: F—TGTTCAACCAATTATCAGCAAACTC; R— TTCTGCTGTATCCTCTTCACCAGT. Actin was amplified using the primer pair F— ATTGGCAATGAGCGGTTC; R—CGTGGATGCCACAGGACT. Real‐time PCR was performed on a ViiA 7 instrument (Thermo Fisher Scientific) using the default two‐step protocol and the data were analyzed in QuantStudio software using the ΔΔ*C*_t_ method. *MBTPS2* expression was normalized to GAPDH (Taqman assays) and BiP expression was normalized to actin (SYBR green).

### 2.7 Immunoblot

Cells were lysed in RIPA buffer (Cell Signaling Technology) with protease and phosphatase inhibitors (Roche). Nuclei were pelleted via centrifugation at 12,000 rpm for 10 min and supernatants were harvested for immunoblot. Protein was quantitated via BCA assay (Thermo Fisher Scientific), and 5 μg of protein was used for each immunoblot. Samples were run on 4%–12% NuPAGE MOPS/SDS gels per the manufacturer's protocol and transferred onto a PVDF membrane. Membranes were blocked in 5% bovine serum albumin and incubated with primary antibody overnight. BiP antibody (BD Bioscience 610979) was used at a concentration of 1:500, rabbit anti‐FLAG antibody (Sigma‐Aldrich SAB4301135) was used at a concentration of 1:500, and beta actin antibody (Santa Cruz sc‐69879) was used at a concentration of 1:200. Secondary antibodies were used at a concentration of 1:1000 and included goat anti‐mouse (Thermo Fisher Scientific 31439) and goat anti‐rabbit (Thermo Fisher Scientific 31462) antibodies. The signal was acquired using SuperSignal West chemiluminescent reagent (Thermo Fisher Scientific) and a KwikQuant digital CMOS imager (Kindle Biosciences).

### 2.8 Immunoprecipitation

Stably transfected M19‐CHO cells were grown in a T75 flask to confluence in DMEM:F12 supplemented with 10% fetal bovine serum and 400 μg/ml G418. Six million cells were lysed in 300 μl of RIPA buffer with protease and phosphatase inhibitors (Roche). MBTPS2 was immunoprecipitated using 40 μl of bead‐conjugated mouse anti‐flag antibody (Sigma‐Aldrich A2220).

### 2.9 Luciferase reporter assays

Luciferase assays were done using a modified version of previously described protocols (Oeffner et al., 2009; Zelenski et al., 1999). Briefly, cells were plated in DMEM:F12 supplemented with 5% lipoprotein‐deficient serum (Sigma‐Aldrich) and 20 μM sodium oleate (Sigma‐Aldrich) in a 96‐well plate at a density of 15,000 cells/well. On Day 1, cells were transfected with a 1:10 mixture of *Renilla* luciferase driven by a constitutive thymidine kinase promoter and firefly luciferase driven by a hamster HMG‐CoA synthase promoter containing a sterol regulatory element (SRE) (gift of Timothy Osborne, Addgene #60444). FuGENE HD (Promega) was used as the transfection reagent according to the manufacturer's protocol. Four hours posttransfection, the media was changed to sterol‐deficient media (DMEM:F12 with 5% lipoprotein deficient serum, 50 μM lithium mevalonate [Sigma‐Aldrich], and 50 μM compactin [Sigma‐Aldrich], or sterol‐enriched media with the addition of 10 μg/ml water‐soluble cholesterol [Sigma Aldrich] and 1 μg/ml 25‐hydroxycholesterol [Sigma‐Aldrich]). After 24 h, luciferase activity was measured using the Dual‐Luciferase Reporter Assay System (Promega) in a SpectraMax L microplate luminometer with reagent injectors (Molecular Devices).

### 2.10 Cell survival assays

Cell survival assays were done as previously described for *MBTPS2* (Oeffner et al., 2009). Briefly, cells stably transfected with pcDNA (control), wild‐type *MBTPS2*, or variant‐*MBTPS2* were plated in a 5.5‐cm dish at a density of 1 × 10^5^ cells in DMEM:F12 supplemented with 10% FBS and 400 μg/ml G418. The following day, media was switched to sterol‐deficient media (DMEM:F12 with 5% lipoprotein‐deficient serum and 400 μg/ml G418) or sterol‐enriched media (DMEM:F12 with 5% lipoprotein‐deficient serum, 5 μg/ml water‐soluble cholesterol, 100 μM sodium mevalonate, 20 μM sodium oleate, and 400 μg/ml of G418). Media was refreshed on Day 3. On Day 6, cells were harvested for quantitation or staining. Cell quantitation was done via counting with a hemocytometer. For staining studies, cells were washed once with 1 × PBS, fixed for 15 min in 4% paraformaldehyde (Thermo Fisher Scientific) in PBS, and then washed once with distilled water. Cells were then stained for 20 min in a 1% crystal violet solution in water (Sigma‐Aldrich). Cells were washed three times in water and dishes were dried overnight. Plate images were obtained via the Epson Perfection V800 Photo scanner.

### 2.11 Tunicamycin experiments

M19‐CHO cells stably transfected with pcDNA (control), wild‐type *MBTPS2*, or variant‐*MBTPS2* were plated in DMEM:F12 supplemented with 10% fetal bovine serum and 400 μ/ml G418 in a 12‐well plate at a density of 150,000 cells/well. On Day 1, media was changed to DMEM:F12 supplemented with 10% fetal bovine serum and 2 μg/ml tunicamycin in DMSO (Sigma‐Aldrich) or equal volume DMSO (control). On Day 2, cells were harvested in Trizol (RNA studies) or RIPA buffer supplemented with protease and phosphatase inhibitors (Roche).

### 2.12 Statistical methods

All experiments were performed in triplicate with three technical replicates per experiment, apart from the immunoprecipitation experiments, which were done five times but without technical replicates, the survival assays, which were done in triplicate but without technical replicates, and the tunicamycin immunoblots, which were done in triplicate but without technical replicates. Data from the *MBTPS2* expression studies and cell survival assays were analyzed via one‐way ANOVA using GraphPad Prism software. Luciferase assay data and quantitative PCR data from the tunicamycin experiments were analyzed via two‐way ANOVA with Dunnett's posthoc test using GraphPad Prism software.

### 2.13 Editorial policies and ethical considerations

All members of the family agreed to participate in this study and signed appropriate consent forms. Permission for clinical photographs was given separately. This study was approved by the institutional IRB (Protocol # 16‐013278).

## 3 RESULTS

### 3.1 Clinical course

**FIGURE 1:**
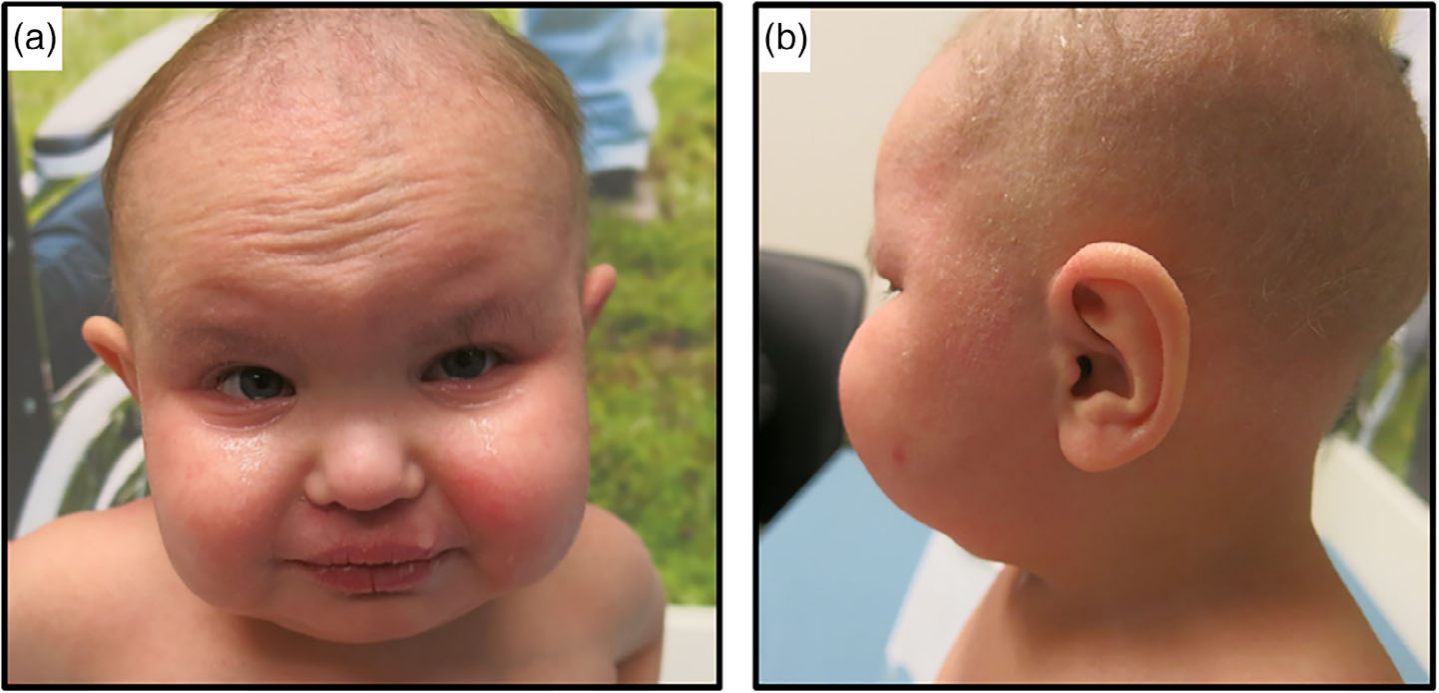
Clinical presentation of the proband. (a) Frontal view at 3 years of age showing dry and erythematous skin, skin exfoliation, sparse and thin scalp hair, sparse eyebrows, prominent ears, and prominent cheeks (b) profile view at 3 years of age showing large ears and dry, scaling skin

Patient was conceived by a 22‐year‐old G1P0➔1 mother. Pregnancy was complicated by prenatal ultrasound findings concerning for cystic kidneys, intrauterine growth restriction, and congenital heart disease. Patient was born at 36‐week gestational age via vaginal delivery. Birth weight was 2.353 kg (25%). He remained in the hospital for the first month of life for management of his atrial septal defect, ventricular septal defect, pulmonic stenosis, and chronic kidney disease (CKD), and for hypospadias repair. Karyotype, chromosomal microarray, and exome sequencing including reanalysis were performed and were negative. His clinical course over months evolved to include microcephaly, sensorineural hearing loss, and gross motor and language delay. His CKD progressed, and he developed macrocytic, hypoproductive anemia, mild thrombocytopenia, and severe developmental delay. At 3 years of age, his growth parameters were notable for a height of 78 cm (<1%; 50% for 15 months), a weight of 10.5 kg (<1%; 50% for 7 months of age), and a head circumference of 44.5 cm (<1%; 50% for 7 months of age). Physical examination was notable for deeply set eyes with normal interpupillary distance, fleshy earlobes, hypotonia, micropenis with undescended testicle on the right, dry and erythematous skin, flaking at the lips, skin exfoliation, sparse and thin scalp hair, and sparse eyebrows (Figure 1a,b). Mitochondrial DNA sequencing for concern for Pearson syndrome was sent and was nondiagnostic. Bone marrow biopsy demonstrated hypercellular marrow with myeloid predominant trilineage hematopoiesis without evidence of sideroblasts on iron stain.

Patient and family were enrolled in the CAG for research‐based exome sequencing. Research exome and subsequent clinical validation was notable for a maternally inherited variant of uncertain significance in *MBTPS2* (c.766G>A; (p.Val256Leu)), consistent with a possible diagnosis of BRESHECK syndrome. Ophthalmology examination was normal with no evidence of photophobia. Lipid profile was notable for a normal total cholesterol (109 mg/dl, normal 45–182 mg/dl), hypertriglyceridemia (289 mg/dl, normal 27–125 mg/dl), low high‐density lipoprotein cholesterol (HDL, 17 mg/dl, normal 35–82 mg/dl), and low low‐density lipoprotein cholesterol (LDL, 34 mg/dl, normal 63–129 mg/dl). Direct LDL was 42 mg/dl (Mayo Clinic Laboratories) and bile acid analysis was normal (Cincinnati Laboratories). Upon direct questioning, the mother endorsed a history of multiple eczematous patches, consistent with random X‐inactivation. There is no other family history of eczema or skin abnormalities. Cascade familial testing for the identified *MBTPS2* variant was declined.

At 4 years of age, growth parameters are notable for a weight of 10.3 kg (<1%; 50% for 7 months of age), a height of 86.4 cm (<1%; 50% for 2 years), and a head circumference of 45.3 cm (<1%; 50% for 7 months of age). Clinical course has been complicated by persistent global developmental delays with absent speech, alopecia totalis, severe skin exfoliation, scaling, and erythema, end‐stage kidney disease with dialysis dependence, transfusion‐dependent anemia and thrombocytopenia, and chronic diarrhea. He was initiated on peritoneal dialysis but transitioned to hemodialysis after an episode of fungal peritonitis.

Evaluation for causes of his hypoproductive anemia (nutritional deficiencies, hemoglobinopathies, and hormonal imbalances) was unremarkable, and his anemia worsened despite increasing erythropoietin and darbopoetin doses. Over the last year, he has required red blood cell transfusions every 4–6 weeks. A repeat bone marrow biopsy at 4 years of age demonstrated significant fibrosis with dysplastic megakaryocytes. Chromosome breakage studies on peripheral blood were initially slightly abnormal with sensitivity to diepoxybutane but not within the range of Fanconi anemia. These were repeated in peripheral blood and fibroblasts and were normal. A comprehensive genetic bone marrow failure panel and telomere analysis were unrevealing. Chromosomal microarray and a hematological genetic panel performed on marrow were notable for a maternally inherited *CBL* variant of uncertain significance (c.1549G>A; (p.Gly517Arg)). Mother has no hematological abnormalities.

Gastroenterology workup for his diarrhea has been notable for recurrent *Clostridioides difficile* infections. The etiology of the repeated infections is unknown. Endoscopic evaluation for very‐early onset inflammatory bowel disease was nondiagnostic. Diarrhea improved following treatment courses for *C. difficile*, though some degree of chronic diarrhea persisted, exacerbating his underlying failure to thrive and malnutrition. A congenital diarrhea gene panel was nondiagnostic. Workup for malabsorption was negative. He subsequently experienced catastrophic bowel ischemia/necrotizing enterocolitis totalis of unclear etiology.

### 3.2 Generation of stably transfected *Mbtps2*‐deficient CHO cells for functional studies

**FIGURE 2:**
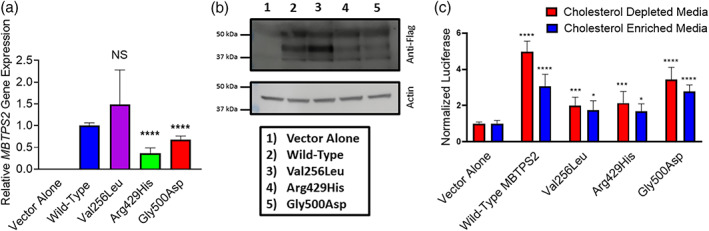
(a) Quantitative PCR demonstrating human *MBTPS2* expression in stable M19‐CHO cell lines. Mutant *MBTPS2* expression levels are normalized to cells expressing wild‐type *MBTPS2*. GAPDH was used as a normalization control. (b) Immunoblot demonstrating S2P protein expression in stable M19‐CHO cell lines (upper panel) and actin normalization control (lower panel). (c) M19‐CHO cells stably transfected with vector alone (negative control), wild‐type *MBTPS2*, or mutation‐bearing *MBTPS2* (Val256Leu, Arg429His, and Gly500Asp) underwent dual transfection with a luciferase reporter gene driven by a sterol‐responsive element and renilla luciferase driven by a constitutive promoter. Results are pooled from three independent experiments. Error bars represent standard deviation. *p < 0.05; **** *p* < 0.0001 by two‐way ANOVA with Dunnett's posthoc test

*Mbtps2* deficiency precludes growth in cholesterol‐deficient media and interferes with proper ER stress pathway activation (Sakai et al., 1996; Ye et al., 2000). To test the ability of our patient‐identified variant to correct these deficiencies in vitro, we generated polyclonal *Mbtps2*‐deficient CHO cells (M19‐CHO) stably expressing vector alone (negative control), wild‐type *MBTPS2* (positive control), Val256Leu‐*MBTPS2* (patient‐identified variant), Arg429His‐*MBTPS2* (BRESHECK syndrome control), and Gly500Asp*‐MBTPS2* (IFAP control) via chemical transfection and G418 selection. Equal or greater expression of the Val256Leu‐*MBTPS2* variant relative to wild‐type *MBTPS2* was confirmed by quantitative PCR and immunoblot (Figure 2a,b). Expression of Arg429His‐*MBTPS2* and Gly500Asp*‐MBTPS2* was 37% and 68% of wild‐type levels, respectively.

### 3.3 The Val256Leu *MBTPS2* variant is unable to activate the SREBP pathway

*MBTPS2* encodes a site‐2 protease (S2P) which activates the transcription factors SREBP1 and SREBP2 via proteolytic cleavage. To determine the ability of mutation‐bearing *MBTPS2* to restore SREBP processing, stably transfected M19‐CHO cells (pcDNA control, wild‐type *MBTPS2*, Val256Leu‐*MBTPS2*, Arg429His‐*MBTPS2*, and Gly500Asp‐*MBTPS2*) were transiently transfected with a luciferase reporter gene driven by a sterol responsive element promoter (Dooley et al., 1998). Cells expressing wild‐type *MBTPS2* were able to process SREBP and induce luciferase gene expression fivefold, whereas cells expressing the *MBTPS2* variants associated with BRESHECK syndrome and IFAP syndrome demonstrated attenuated induction of twofold and 3.5‐fold, respectively. Supplementation of the media with cholesterol produced only a threefold induction of luciferase expression in cells expressing wild‐type *MBTPS2*, consistent with the known physiology of SREBP (Figure 2c).

### 3.4 The Val256Leu *MBTPS2* variant is unable to proliferate in cholesterol‐deficient media

**FIGURE 3:**
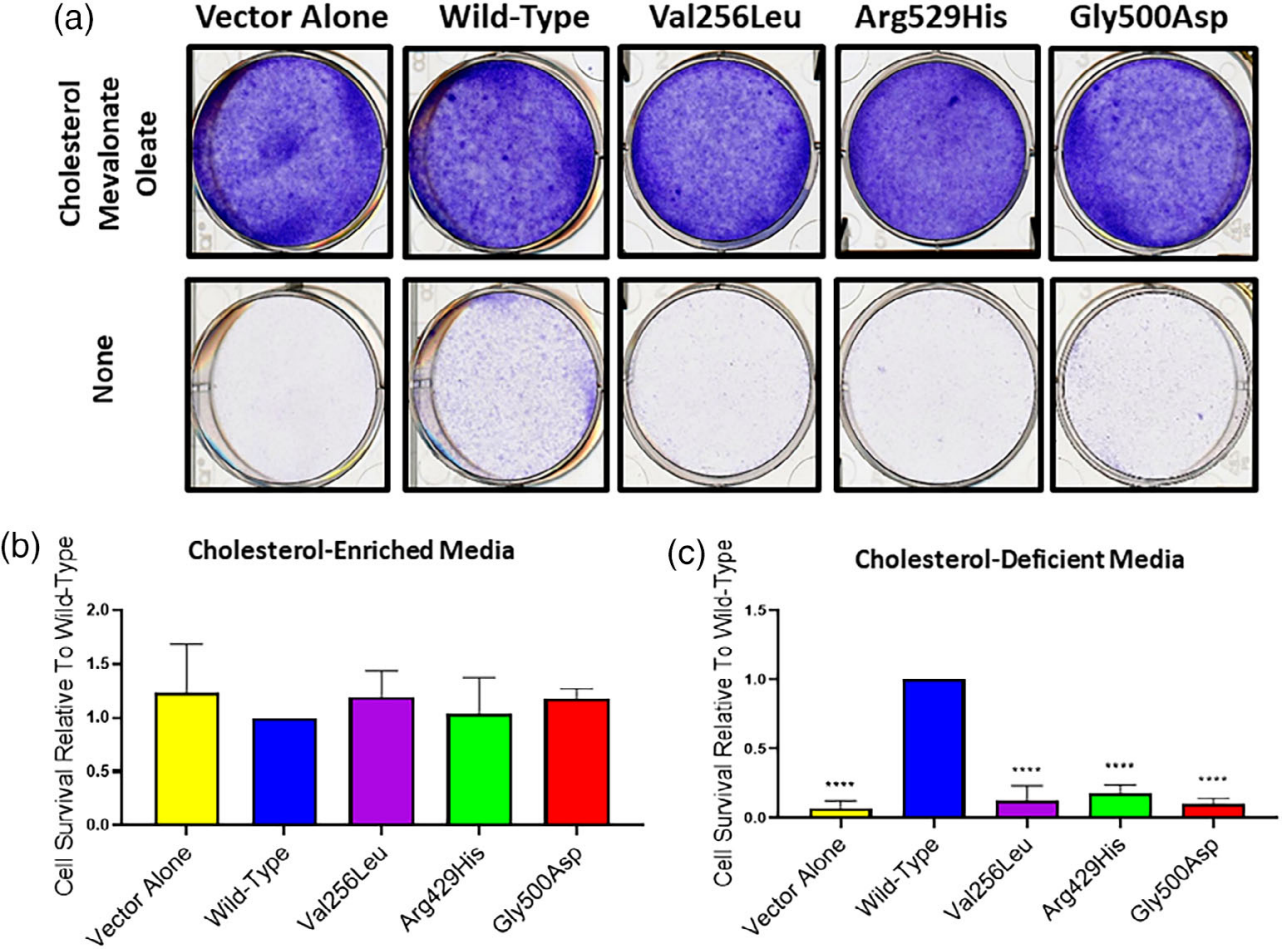
Cells expressing mutant‐*MBTPS2* show diminished viability in cholesterol‐deficient media. (a) M19‐CHO cells were grown in cholesterol‐enriched media (upper panel) or lipoprotein‐ and cholesterol‐depleted media (lower panel) and visualized by crystal violet staining. (b,c) M19‐CHO cells were treated as above and counted via hemocytometer. Cellular counts for each experiment (*n* = 3) were normalized to wild‐type *MBTPS2*‐expressing cells. Error bars represent standard deviation. ****p < 0.0001 by one‐way ANOVA

M19‐CHO cells are unable to grow in cholesterol‐deficient media due to impaired SREBP processing and inability to upregulate de novo cholesterol biosynthesis and cholesterol capture pathways. To determine the ability of mutation‐bearing *MBTPS2* to induce SREBP pathway activation and enable growth in cholesterol‐deficient media, stably transfected M19‐CHO cells (pcDNA control, wild‐type *MBTPS2*, Val256Leu‐*MBTPS2*, Arg429His‐*MBTPS2*, and Gly500Asp‐*MBTPS2*) were grown in cholesterol‐enriched or cholesterol depleted media and survival was assessed by cell staining and cell counting. Expression of wild‐type *MBTPS2* permitted survival in cholesterol‐depleted media, whereas cells expressing mutant‐*MBTPS2* were unviable. All cell lines demonstrated equal growth capacity in media supplemented with cholesterol (Figure 3a–c).

### 3.5 The Val256Leu *MBTPS2* variant demonstrates impaired ER‐stress pathway activation

**FIGURE 4:**
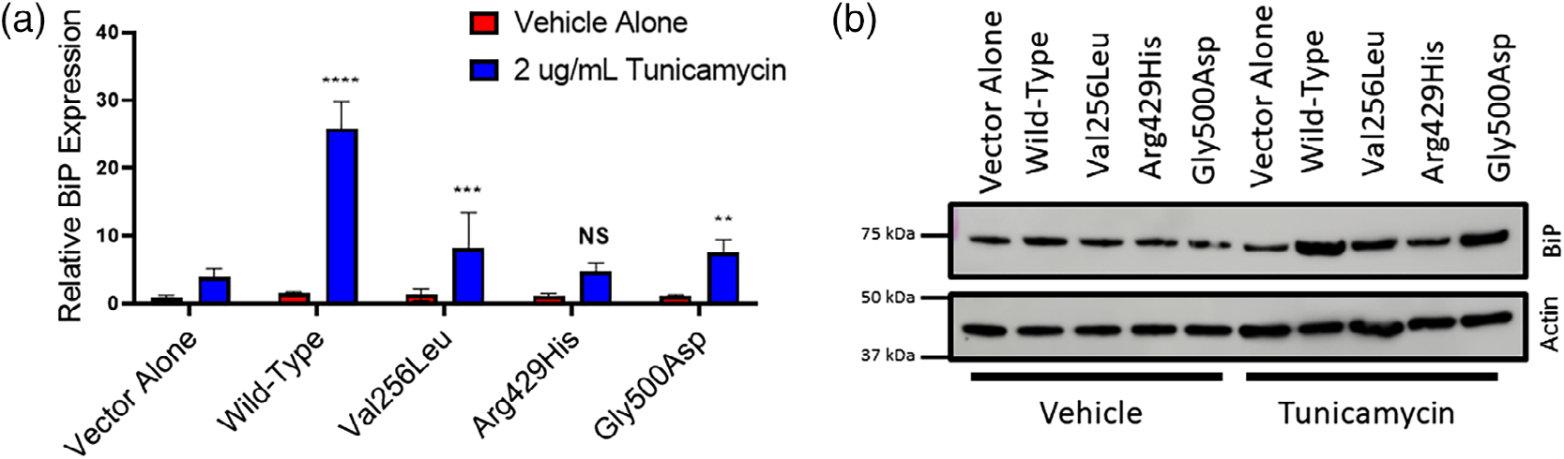
Cells expressing mutant‐*MBTPS2* have reduced ER stress pathway activation. M19‐CHO cells were treated with 2 μg/ml of tunicamycin to induce ER stress. (a) Real‐time PCR demonstrating blunted upregulation of BiP expression with tunicamycin treatment in M19‐CHO cells expressing variant‐*MBTPS2* compared to cells expressing wild‐type *MBTPS2*. Actin was used as a normalization control. Data pooled from three independent experiments, statistical significance analyzed by two‐way ANOVA with Dunnett's posthoc test. (b) Immunoblot demonstrating blunted upregulation of BiP protein with tunicamycin treatment in mutant cells. Representative immunoblot from three independent experiments. Error bars represent standard deviation. **p < 0.01; ***p < 0.001; ****p < 0.0001

S2P activates activation transcription factor 6 (ATF6) as part of the ER stress response through proteolytic cleavage, which generates a transcription factor that increases expression of ER chaperones, including BiP (Haze et al., 1999; Ye et al., 2000). To assess the ability of mutant‐*MBTPS2* to restore ATF6 cleavage, stably transfected M19‐CHO cells (pcDNA control, wild‐type *MBTPS2*, Val256Leu‐*MBTPS2*, Arg429His‐*MBTPS2*, and Gly500Asp‐*MBTPS2*) were treated with tunicamycin to induce ER stress. Upregulation of ER chaperones was assessed by quantitative PCR and immunoblot. Wild‐type *MBTPS2* was able to induce BiP/Grp78 mRNA expression 26‐fold, whereas *MBTPS2* variants associated with BRESHECK syndrome and IFAP syndrome demonstrated attenuated induction of 4.7 to 8‐fold. Mutant‐*MBTPS2* was also impaired in its ability to increase BiP protein (Figure 4a,b).

## 4 DISCUSSION

Here, we report a patient with BRESHECK syndrome with the novel features of chronic diarrhea, anemia, bone marrow fibrosis, hypertriglyceridemia, hypobetalipoproteinemia, and hypoalphalipoproteinemia found by research‐based exome sequencing to harbor a novel Val256Leu *MBTPS2* variant. Similar to the previously reported Arg429His mutant, we show that the Val256Leu mutant has severely compromised function, with impaired ability to activate the SREBP and ER stress pathways (Oeffner et al., 2009).

**FIGURE 5:**
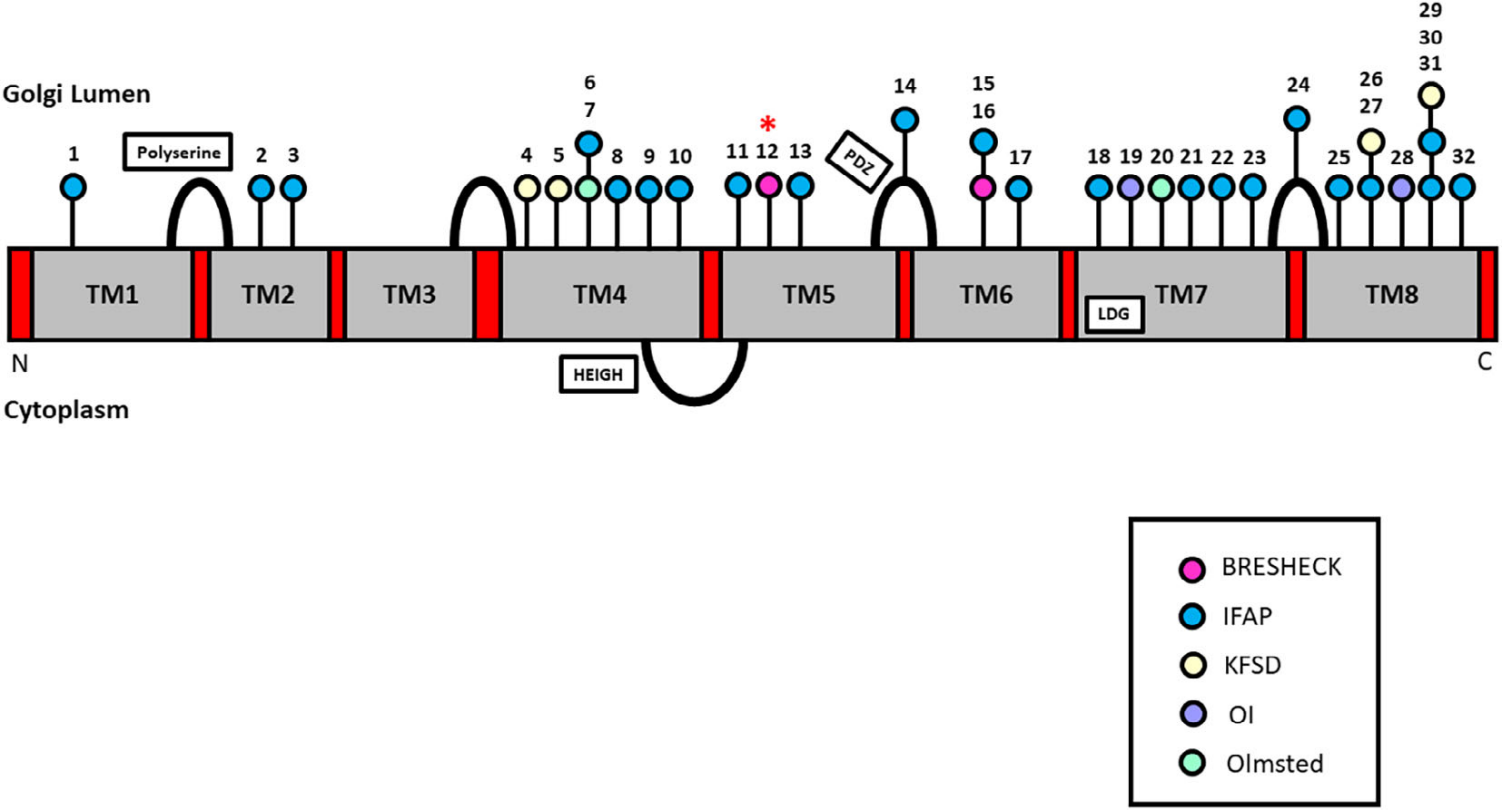
Schematic of the MBTPS2 protein and localization of previously reported variants. The novel Val256Leu variant identified in this study is denoted with an asterisk. PDZ domain: facilitates substrate recognition after S1P cleavage; HEIGH domain: zinc‐binding catalytic domain; LDG: zinc‐binding catalytic domain. IFAP, ichthyosis follicularis atrichia photophobia; KFSD, Keratosis follicularis spinulosa decalvans; OI, osteogenesis imperfecta

The mechanism by which the Val256Leu and Arg429His variants cause severe disease is unknown, but is suspected to be a result of severely impaired S2P catalytic function. The two catalytic domains HEIGH and LDG localize to the cytosolic and seventh transmembrane domains, whereas the Val256Leu and Arg429His variants localize to the fifth and sixth transmembrane domains (Caengprasath et al., 2021; Figure 5). It is possible that the BRESHECK‐associated variants localize to regions critical for proper membrane topology and MBTPS2 function, or that the amino acid substitutions associated with BRESHECK syndrome are more disruptive to MBTPS2 structure and function compared to IFAP‐associated variants. It is also possible that the variants associated with BRESHECK syndrome interfere with S2P stability or localization; however, this is not supported by previous studies (Lindert et al., 2016; Oeffner et al., 2009).

Interestingly, our patient's variant is very close to two previously reported variants identified in IFAP patients without the structural features of BRESHECK syndrome, c. 758G>C; (p.G253A) and c.774C>G; (p.I259M) (Bornholdt et al., 2013). The basis for these disparate phenotypes is unknown. Further characterization of the effects of all identified *MBTPS2* pathogenic variants on the ability of MBTPS2 to activate the SREBP and ER stress signaling pathways would be invaluable in understanding how different variants cause disease, as would protein modeling or a crystal structure of the human MBTPS2 protein.

It is unclear which elements of the BRESHECK phenotype are driven by impaired activation of sterol‐responsive genes and which reflect compromised ER stress pathway activation. Cholesterol is a developmental morphogen that plays a critical role in hedgehog signaling (Huang et al., 2016). Smith–Lemli–Opitz syndrome is an autosomal recessive disorder of cholesterol biosynthesis, and shares many features with BRESHECK syndrome, including microcephaly, developmental delay with seizures, hearing loss, growth failure, congenital heart disease, genitourinary malformations, cleft palate, and photosensitivity (Porter, 2008). Many disorders of cholesterol metabolism are also associated with ichthyosis and abnormal skin barrier function (Herman, 2003). Mendelian disorders characterized by perturbed ER stress response include Wolfram syndrome, Aicardi–Goutieres syndrome, Harlequin ichthyosis, and Wolcott–Rallison syndrome, which can be associated with hearing loss, developmental delay, growth failure, and ichthyosis (Dombroski et al., 2010). The features of BRESHECK syndrome therefore likely reflect a combination of impaired ER stress response and impaired hedgehog signaling from compromised cholesterol biosynthesis.

The lipid profile of BRESHECK syndrome is poorly characterized, though one report suggests a normal lipoprotein profile (Aten et al., 2010) and another reported low HDL only (Kondo et al., 2018). Our patient demonstrated persistently low LDL and HDL and elevated triglycerides. Low LDL likely reflects impaired SREBP‐mediated activation of the rate limiting enzyme in cholesterol biosynthesis, HMG‐CoA reductase. The mechanism by which *MBTPS2* deficiency would cause low HDL‐C is unclear. There is suggestion that SREBP can induce endothelial lipase and scavenger receptor B1 gene expression, both genes critical in HDL metabolism; however, the biology of these genes would suggest that *MBTPS2* deficiency would increase HDL levels (Kivelä et al., 2012; Lopez & McLean, 1999). Single‐nucleotide polymorphisms near *MBTPS2* have been associated with HDL‐C in women (Lu et al., 2008). The mechanistic basis of our patient's hypertriglyceridemia is similarly unknown. It is possible that our patient's HDL and triglyceride phenotype reflects his underlying kidney disease, which is known to cause hypertriglyceridemia and hypoalphalipoproteinemia (Sethna et al., 2019).

In addition to the typical skin and organ dysfunction seen in BRESHECK syndrome, our patient additionally presented with chronic diarrhea, cytopenias, and bone marrow fibrosis. Diarrhea is a common feature in disorders of cholesterol metabolism including lysosomal acid lipase deficiency, abetalipoproteinemia, cerebrotendinous xanthomatosis, and Niemann–Pick disease. Importantly, the etiology in these diseases relates to cholestasis, impaired bile acid metabolism, and the inability to efficiently assemble and absorb chylomicrons and consequent steatorrhea, which was not present in our patient. Bone marrow fibrosis is not typical of disorders of cholesterol metabolism. These phenotypes may reflect compromised ER stress responses, as both intestinal epithelium and bone marrow cells are highly proliferative and experience high protein load in the ER secretory pathway (Sharma et al., 2019). Our patient's novel *MBTPS2* variant may more severely compromise *MBTPS2* function compared to previously reported variants, accounting for our patient's more severe phenotype. A functional difference may not have been apparent in our in vitro studies due to assay sensitivity or due to the unequal expression of the Val256Leu and Arg429His variants in our in vitro system. A more complete molecular characterization of these variants would be required to understand the varied clinical phenotypes.

In summary, we present a patient with the clinical stigmata of BRESHECK syndrome with the additional features of bone marrow fibrosis, chronic diarrhea, and dyslipidemia. Our case identifies a novel variant for BRESHECK syndrome and demonstrates its deleterious effect on gene function, with impaired activation of sterol‐dependent transcription and the ER stress response.

## CONFLICT OF INTERESTS

The authors declare that there are no conflict of interests.

## AUTHOR CONTRIBUTIONS

Alanna Strong and Hakon Hakonarson conceptualized and designed the study, participated in data collection and analysis, and drafted the manuscript. Christopher J. Cardinale, Jeffrey Billheimer, Michael E. March, Sophia E. Kim, Deborah Watson, Courtney Vaccaro, Cuiping Hou, and Diana Slater participated in experimental design, data collection and analysis, and critically reviewed the manuscript. Jamie Merves, Hilary Whitworth, Leslie Raffini, Christopher Larosa, Lawrence Copelovitch, and Elaine H. Zackai evaluated the patient clinically and critically reviewed the manuscript. All authors approved the final manuscript as submitted and agree to be accountable for all aspects of the work.

## Data Availability

N/A
